# Atomically controlled substitutional boron-doping of graphene nanoribbons

**DOI:** 10.1038/ncomms9098

**Published:** 2015-08-25

**Authors:** Shigeki Kawai, Shohei Saito, Shinichiro Osumi, Shigehiro Yamaguchi, Adam S. Foster, Peter Spijker, Ernst Meyer

**Affiliations:** 1 Department of Physics, University of Basel, Klingelbergstrasse 82, CH-4056 Basel, Switzerland; 2 PRESTO, Japan Science and Technology Agency, 4-1-8, Honcho, Kawaguchi, Saitama 332-0012, Japan; 3 Department of Chemistry, Graduate School of Science, Nagoya University, Furo, Chikusa, Nagoya 464-8602, Japan; 4 Institute of Transformative Bio-molecules, Nagoya University, Furo, Chikusa, Nagoya 464-8602, Japan; 5 CREST, Japan Science and Technology Agency, 4-1-8, Honcho, Kawaguchi, Saitama 332-0012, Japan; 6 COMP, Department of Applied Physics, Aalto University, PO Box 11100, FI-00076 Helsinki, Finland

## Abstract

Boron is a unique element in terms of electron deficiency and Lewis acidity. Incorporation of boron atoms into an aromatic carbon framework offers a wide variety of functionality. However, the intrinsic instability of organoboron compounds against moisture and oxygen has delayed the development. Here, we present boron-doped graphene nanoribbons (B-GNRs) of widths of *N*=7, 14 and 21 by on-surface chemical reactions with an employed organoboron precursor. The location of the boron dopant is well defined in the centre of the B-GNR, corresponding to 4.8 atom%, as programmed. The chemical reactivity of B-GNRs is probed by the adsorption of nitric oxide (NO), which is most effectively trapped by the boron sites, demonstrating the Lewis acid character. Structural properties and the chemical nature of the NO-reacted B-GNR are determined by a combination of scanning tunnelling microscopy, high-resolution atomic force microscopy with a CO tip, and density functional and classical computations.

## Abstract (web summary)

Incorporation of boron atoms into an aromatic carbon framework offers a wide variety of functionality. Here, the authors present boron-doped graphene nanoribbons by on-surface chemical reaction and characterize the structures and properties using scanning probe microscopy at the atomic-scale.

## Graphical abstract

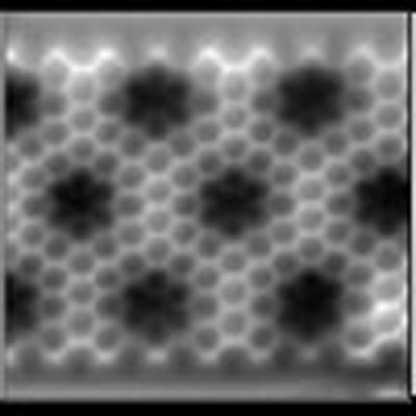

Boron is a unique element in terms of electron deficiency and Lewis acidity, yet it has a comparable size to carbon. Incorporation of boron atoms into an aromatic carbon framework like carbon nanotubes^1^ and graphene^2^ offers a wide variety of functionality in, for example, chemical sensing^3^,^4^, nanoelectronics^5^,^6^, photocatalysis^7^ and battery electrodes^8^. So far, the syntheses have been demonstrated by both the chemical vapour deposition method with co- or mono-depositing of small molecules, like B_2_H_6_, BCl_3_, and PhB(OH)_2_ (refs 7, 8, 9, 10, 11), and by the reactive microwave plasma method with B(CH_3_)_3_ (ref. 12). However, besides the formation of Stone–Wales defects,^10^ the coordination environment of the boron sites in the graphitic carbons, namely the doping site and density of boron, cannot be precisely controlled in these methods. This limitation can be overcome by a recently developed on-surface chemical reaction^13^,^14^. In fact, nitrogen atoms have been doped into the edges of graphene nanoribbons (GNRs) by employing nitrogen-doped precursor molecules^15^,^16^. In contrast, the intrinsic instability of organoboron compounds against moisture and oxygen has delayed the development of boron-doped nanocarbon chemistry^17^,^18^ and has led to the unavailability of boron-doped precursor molecules. However, our recent chemical synthesis of polycyclic aromatic hydrocarbons with boron atoms proved the stability of this materials class^19^. The use of such an organoboron precursor can pave the way for boron doping into GNRs. We now present a well-structured boron-doped-GNR (B-GNR) in terms of valency, position and doping ratio and study its chemical structure and property by high-resolution atomic force microscopy (AFM) and scanning tunneling microscopy (STM).

## Results

### Synthesis of boron-doped graphene nanoribbons

**Figure 1:**
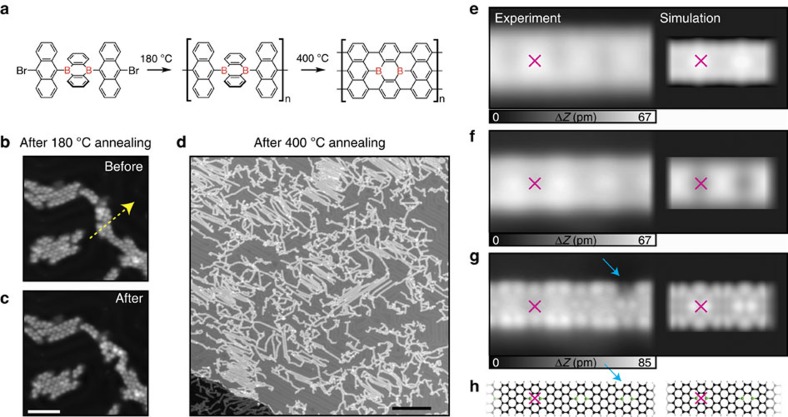
Synthesis of boron-doped graphene nanoribbons. (**a**) Schematic drawing of the on-surface chemical reaction. (**b**) STM of molecules, polymerized by annealing at 180 °C and (**c**) after the tip-induced manipulation. Scale bar, 5 nm. (**d**) B-GNR, synthesized by annealing at 400 °C. Scale bar, 30 nm. (**e**–**g**) High-resolution STM topographies (left) and corresponding simulated STM images (right) of B-GNR, taken with different bias voltages. (**h**) Chemical structure of B-GNR. Black and pink balls indicate carbon and boron atoms. Measurement parameters: tunnelling current *I*=5 pA and bias voltage applied to the tip *V*=−200 mV for (**b**–**d**), *I*=10 pA and *V*=−3 V for (**e**), *I*=10 pA and *V*=3 V for (**f**), and *I*=1 nA and *V*=−2 mV for (**g**).

Figure 1a shows the chemical structure of the precursor molecule, in which two pristine anthracene units and one 9,10-dibora-9,10-dihydroanthracene moiety are covalently bonded while two bromine atoms are located at both termini. By annealing the molecules on a Au surface at 180 °C, the bromine atoms are removed by surface catalytic dehalogenation. Consequently, the precursor molecules are linearly polymerized. At an elevated temperature of up to 400 °C, the surface-assisted cyclodehydrogenation results in the formation of an aromatic graphene nanoribbon^20^. Employing this reaction, boron atoms can be doped as BC_3_ Lewis acidic sites at the centre row in the armchair-edge GNR (B-GNR) with a width of seven carbon atoms (*N*=7). The interval between the boron sites is fixed with a doping density of 4.8 atom%.

In experiments, the precursor molecules were deposited on a clean Au(111) surface in ultra-high vacuum and were subsequently annealed at 180 °C (Fig. 1b). At 4.8 K the molecules mainly stay along the valley of the herringbone structure on Au(111). We performed tip-induced lateral manipulation to check the presence of the polymer chain. After scanning the tip at a constant height along the line indicated with an arrow in Fig. 1b, several molecular units moved together, indicating successful polymerization (Fig. 1c). The successive anthracene units have opposite tilts to the Au substrate owing to the repulsion between hydrogen atoms in the adjacent anthracene moieties. After annealing at 400 °C, such corrugated structures disappeared. Instead, stripe-shaped flat nanoribbons appeared (Fig. 1d).

The observed STM contrast is inverted by the polarity of the applied tip bias voltage (−3 and 3 V), indicating the perturbation of the electronic structure by the doped boron atoms as an electron-accepting unit (Fig. 1e,f, Supplementary Fig. 1). Pink crosses indicate the same atomic site in B-GNR. By setting close to zero bias voltage (−2 mV), the electronic states near the Fermi level of B-GNR are resolved (Fig. 1g). We also observed two different contrasts at the armchair type edge, where the ratio of one dark- and two bright-contrasts suggests that the dark-contrast edge relates to the boron-doped anthracene. The hydrogen atoms at the armchair edge can be desorbed by the tunnelling current at a large negative tip bias (−3.5 V and 100 pA, Supplementary Fig. 2) as indicated with an arrow. The simulated STM topographies (right panel in Fig. 1e–g) are in good agreement with the experimental results (left panel), so that the chemical structure can be unambiguously assigned as shown in Fig. 1h.

### Electronic structure of boron-doped graphene nanoribbons

**Figure 2:**
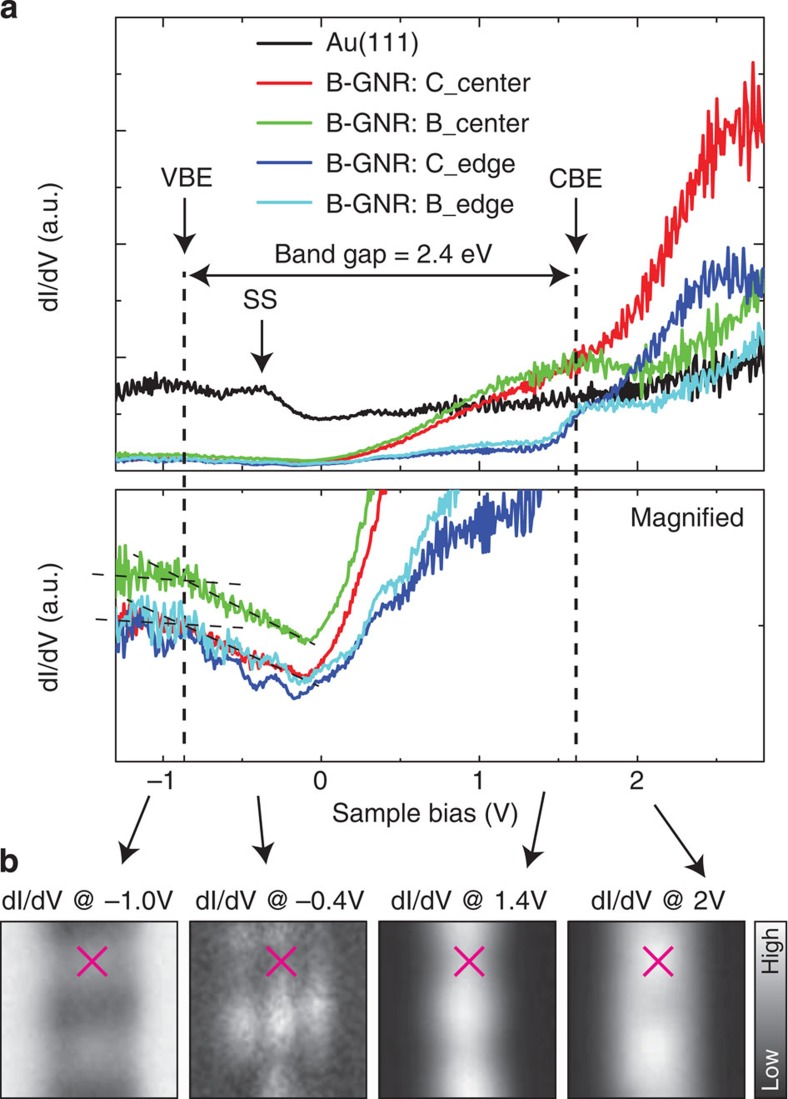
d*I*/d*V* curve on B-GNR. (**a**) Differential conductance (d*I*/d*V*) spectra taken at four different sites of *N*=7 B-GNR and one Au(111) site with a clean Au tip. The spectra were numerically calculated from the measured bias dependent curves of the tunnelling current. The bias voltage is only here redefined with respect to the tip grounding instead of the sample grounding. (**b**) Constant height d*I*/d*V* maps measured with a lock-in amplifier (root mean square amplitude=14 mV and frequency=521 Hz) at the different bias voltages. Pink crosses indicate at the centre of the same C_4_B_2_ site.

To measure the electronic structure of *N*=7 B-GNR, d*I*/d*V* spectra were taken with a clean Au tip above four different sites of B-GNR and, for reference, above one Au(111) site (Fig. 2a). The conductance is related to the local density of states at the tip position. At the Au(111) site, the effect of Shockley surface states locates around −0.5 eV below the Fermi energy. The contribution, originating from the surface state, is even visible on B-GNR as modulations of the d*I*/d*V* spectrum down to −1.0 eV, and rather stronger than on the non-doped GNR^21^,^22^,^23^. This may relate to the stronger coupling to the substrate due to the presence of boron atoms. More interestingly, in contrast to the pristine GNR^22^, the presence of these boron atoms downshifts the interface state. Nevertheless, the valence band edge locates around −0.8 eV below the Fermi energy, with clear peaks at 1.6 eV corresponding to the conduction band edge. Consequently, the band gap of the B-GNR is 2.4 eV, which is comparable to the non-doped GNR on Au(111) (ref. 22). Previous theoretical studies indicate that the position of doped boron strongly affects the modulation of the electronic structure and so that the minimum (maximum) effect is expected with a doping at the centre (edge)^5^. In this system, boron atoms locate exactly at the centre of *N*=7 B-GNR and, thus, no significant electron–hole asymmetry is present (full suppression of backscattering), leaving intact the overall electronic structure (this is seen also in our calculated electronic structure, Supplementary Fig. 3). Yet, in a detailed inspection, the presence of boron atom in the GNR can be observed. The inversions of the conductance between the doping and non-doping sites occur twice between 1.4 and 2.0 eV and between −1.0 and −0.4 eV both at the edge and centre. This is consistent with the scanning tunnelling spectroscopy (STS) images taken within the energy levels at constant heights (Fig. 2b), and also the location of the boron features in the calculated projected density of states.

### Fused boron-doped graphene nanoribbons

**Figure 3:**
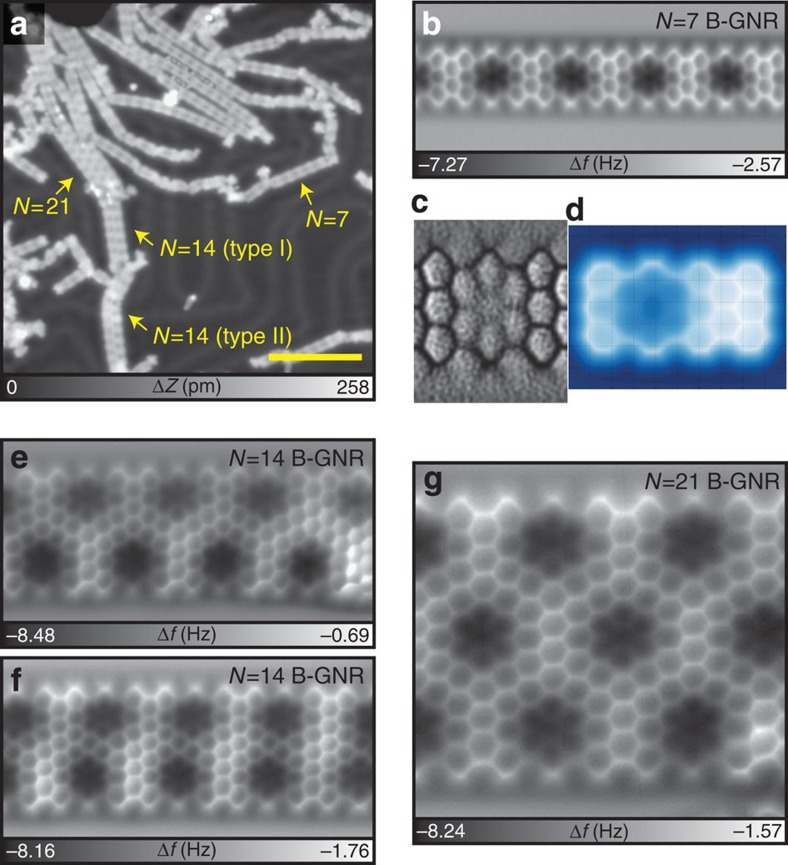
Fused B-GNR. (**a**) STM overview of fused B-GNR. Scale bar, 10 nm. (**b**) Frequency shift Δ*f* map of *N*=7 B-GNR and (**c**) the corresponding Laplace filtered image for a better view of bonds. (**d**) and the simulated AFM image. (**e**,**f**) Δ*f* maps of fused *N*=14 B-GNR with different structures. (**g**) Δ*f* map of fused *N*=21 B-GNR. Measurement parameters: *A*=38 pm and *V*=0 V.

After the substrate was further annealed at 510 °C, B-GNRs with different widths (*N*=7, 14 and 21) were obtained by fusing the armchair edges (Fig. 3a). To check the chemical structure in detail, we used an AFM with a CO-functionalized tip^24^. In this technique, when the tip-sample separation is small enough, the tip detects the short-range Pauli repulsive interaction^25^ and the bond structure of molecules can be resolved via the frequency shift of the oscillating force sensor^24^,^26^,^27^,^28^. Figure 3b shows the AFM image of *N*=7 B-GNR obtained in a constant height mode. The periodic aromatic carbons are clearly revealed, in which every three anthracene units have a defect-like feature, with darker contrast (more negative frequency shift). To enhance the observed bond visually, a Laplace filter^27^ was applied in the region around the darker contrast site (Fig. 3c). The C–C bonds in the anthracene unit are clearly observed and, even at the centre, the B–C bonds were observed.

Although the AFM imaging mechanism with a CO tip is somewhat sensitive to the total density of charge^25^, the nature of the contrast cannot fully be explained by the inherent electron-deficient nature of boron and its electron-accepting character. Thus, we consider the topographic geometry in detail. Although the van der Waals radius of the B atom (192 pm) is larger than the C atom (170 pm), the actual topographic shift of the boron atom core in B-GNR on Au obtained by our density functional theory (DFT) calculation is a 30 pm dip (Supplementary Fig. 4), which clearly plays an important role in the more negative frequency shift observed and predicted in simulations (see also calculated the AFM image in Fig. 3d and Supplementary Fig. 5). In the absence of this topographic distortion, the difference in local charge density and resulting electrostatic interactions is not enough to produce a clear contrast change. The B–C bond (longer than the C–C bond) also deforms the honeycomb structure^19^, while the distortion of the carbon ring in the AFM image relates to the effect of tilting CO tip^27^.

On the basis of these observations, we state that the on-surface chemical reaction was accomplished as demonstrated in Fig. 1a. Some defects, presumably relating to the on-surface dissociation of B–C bonds in the first annealing process, are also observed (Supplementary Figs 6 and 7). We further analysed B-GNRs with wider widths (Supplementary Fig. 8). For *N*=14 B-GNRs, two different fused configurations with the out-of-phase (Fig. 3e) and the shifted phase (Fig. 3f) were observed. Since the configuration is determined by the relative position in the fusing process, the shifted phase *N*=14 B-GNR has a mirror configuration, thus, in total three configurations. In such a process, the C–C link seems to be caused by a zipping up process through dissociating hydrogen atoms at the armchair edge (Supplementary Fig. 9). For *N*=21 B-GNRs, nine different textures are theoretically expected. Among them, the perfectly ordered out-of-phase structure is shown in Fig. 3g. The boron atoms can stay at the designated atomic site throughout the annealing process at the higher temperature of 510 °C. Such high stability may reflect the robustness of the BC_3_ site^29^. Therefore, once a boron atom is perfectly incorporated in graphene, the thermal stability is remarkably high (Supplementary Fig. 10).

### Lewis acidity of the boron site

**Figure 4:**
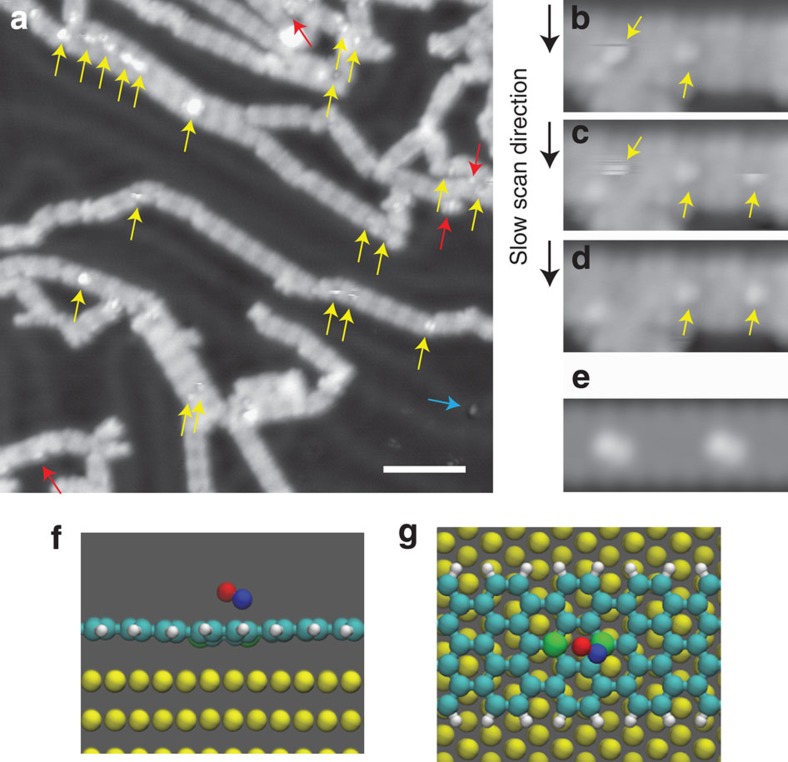
NO adsorption. (**a**) STM topography of B-GNR with NO molecules. Blue, red and yellow arrows indicate NO molecules attached at the elbow of herringbone structure on Au(111), the armchair edge, and the boron site of the B-GNRs, respectively. Scale bar, 5 nm. (**b**–**d**) A series of STM images to show the hopping NO between the boron sites. (**e**) Simulated STM image. (**f**,**g**) Side and top views of calculated conformation of NO adsorbed at the boron site in B-GNR. Nitrogen, oxygen and boron atoms are shown in blue, red and green, respectively. Measurement parameters: *V*=−200 mV and *I*=1 pA in (**a**) and *I*=2 pA in (**b**–**d**).

Since the structural and electronic characters of the BC_3_ sites were demonstrated, we next evaluate the Lewis acidity of the boron sites by exposing the B-GNRs to nitric oxide (NO) gas. Figure 4a shows a large-scale STM image, where small dots correspond to dosed NO molecules. It is well-known that the NO molecules adsorb at the elbow site of the Au(111) herringbone structure (blue arrow)^30^. More preferentially, NO molecules attach to the edge of the B-GNR (red arrow). However, it is more important that the NO molecules were most efficiently trapped at the centre of the B-GNR (yellow arrow), corresponding to the boron site. By setting the tip closer to the sample surface, this NO molecule can be easily manipulated by the scanning tip. Figure 4b–d present a series of the STM topographies, showing the movement of NO. The half-moon-like feature in Fig. 4c corresponds to the manipulated NO by the scanning local probe. Since the NO molecule showed a hopping behaviour on specific boron sites, the BC_3_ moieties exhibit a Lewis acidic role for the stable gas adsorption. The simulated STM image (Fig. 4e) with optimized adsorption model (Fig. 4f,g) shows that the NO molecule is bound to the B site via the N atom. The calculated binding energy of 1.5 eV is ideal for a chemical sensor, since a continuous adsorption at room temperature is expected to show a linear response to the NO density. The NO chemical adsorption significantly changes the electronic structure of the B-GNR, introducing new states into the band gap (Supplementary Fig. 3), which suggests a strong electric response in a device. To refresh the device and avoid saturation, a small increase in thermal energy will cause desorption.

## Discussion

We present a programmed synthesis of boron-doped graphene nanoribbons with uniform texture of Lewis acidic site. Ullmann type on-surface polymerization and the following thermal cyclodehydrogenation reactions were employed for a triarylborane precursor with an elaborate design. The uniform BC_3_ sites are doped at designated positions with a fixed doping ratio according to the precursor. Namely, two boron atoms exist at the centre row in every three anthracene moieties and the doping density is 4.8 atom%. The chemical structures of B-GNRs with different widths of *N*=7, 14 and 21 were directly resolved by high-resolution force microscopy with a CO tip. Lewis acidity of the electron-deficient boron sites is experimentally demonstrated by nitric oxide adsorption at thermal equilibrium. This behaviour proposes a new approach for temperature-dependent dynamic perturbation of the electronic properties of B-GNRs around the Fermi level in an NO atmosphere, while the previously reported structural modifications, by varying the width of the pristine GNR^31^ or by nitrogen atom doping of the edge^15^,^16^, have realized only static control. The tunable response presented here offers great potential in many technologies, particularly where dynamic control would be a disruptive development. Besides this application, the fabrication of boron-doped nanoribbons could allow for the design of mobility gaps and novel types of graphene transistors^32^,^33^. In the *N*=7 B-GNR, the boron locates exactly at the centre of the ribbon, so that the backscattering is fully suppressed. Yet, in the fused B-GNRs, the symmetry can vary. Further, the asymmetry can be increased by co-depositing boron-doped and non-doped precursor molecules. To fabricate such devices, transferring on-surface synthesized B-GNRs is a critical issue, yet we believe that the recently developed thin film etching technique can solve this^16^,^34^.

## Methods

### Experimental measurement

All experiments were performed with an Omicron STM/AFM with a qPlus configuration^35^, operating at 4.8 K in ultrahigh vacuum. A clean Au(111) surface was *in-situ* prepared by repeated cycles of standard sputtering and annealing. The tungsten tip of a tuning fork sensor was ex-situ sharpened by a focused ion beam milling technique and was then *in-situ* covered with Au atoms by contacting to the sample surface. The resonance frequency of the self-oscillating qPlus sensor was detected by a digital PLL circuit (Nanonis: OC4 and Zurich Instruments: HF2LI and PLL). 9,10-bis(10-bromoanthracen-9-yl)-9,10-dihydro-9,10-diboraanthracene was deposited on the substrate from a crucible of Knudsen cell, resistively heated at 240 °C. Subsequently, the sample was annealed at 180 and 400 °C to synthesize the *N*=7 B-GNR. The fused B-GNRs with *N*=14 and 21 were obtained by further annealing at 510 °C. Further, NO gas was introduced into the ultrahigh vacuum chamber (100 Langmuir) while the sample stayed in the cold microscope. In STM mode, the tip was biased while the sample was electronically grounded. The topographic images were taken in a constant current mode. In AFM mode, the tip apex was terminated by a CO-molecule and all images were taken at a constant height mode.

### Synthesis of precursor molecule

The precursor **1**, 9,10-bis(10-bromoanthracen-9-yl)-9,10-dihydro-9,10-diboraanthracene, was obtained from compounds **2** and **3** through the synthetic route shown in Supplementary Fig. 11a. Although some derivatives of the precursor **1** with extra bulky substituents have been reported^36^, the precursor **1** has not been characterized because of its low solubility in common organic solvents. We successfully characterized the isolated precursor **1** by the NMR analyses in tetrachloroethane at 100 °C and by X-ray crystal structure analysis using chlorobenzene as solvent. The crystalline sample of the precursor **1** can be stored at ambient conditions as a result of the steric protection of the boron atoms by the anthracene groups. Compounds **2** (ref. 37) and **3** (ref. 38) were prepared according to literature procedures. The compound **3** was stored in a glove box due to the moisture sensitivity. All reactions were carried out under a nitrogen atmosphere. Commercially available solvents and reagents were used as received. Dry ether and *n*-BuLi (1.6 M in hexane) were purchased from Wako Pure Chemical Industries. Dry toluene was purchased from Kanto Chemical.

### Precursor molecule 1

To a solution of **2** (231 mg, 0.605 mmol) in ether (13 ml) cooled at 0 °C was added *n*-BuLi (1.6 M in hexane, 0.378 ml, 0.605 mmol) dropwise. The mixture was stirred at room temperature for 20 min. After removing the solvent *in vacuo*, the residue was dissolved together with **3** (101 mg, 0.303 mmol) in toluene (8 ml). The resulting mixture was stirred at room temperature for 16 h, and the solvent was removed *in vacuo*. The crude product was washed with MeOH and hexane and further purified by silica gel column chromatography using CHCl_3_/hexane (ratio 2:1) as an eluent. Recrystallization from chlorobenzene afforded **1** as an orange solid (31.2 mg, 45.5 μmol, 15%). Thin layer chromatography (CHCl_3_/hexane, 2:1 v/v): *R*_f_=0.90; ^1^H-NMR (600 MHz, 1,1,2,2-tetrachloroethane-*d*_2_, 100 °C): δ 8.72 ppm (d, *J*=9.0 Hz, 1H), 7.77 (d, *J*=8.4 Hz, 1H), 7.68 (dd, *J*_1_=9.0 Hz, *J*_2_=7.8 Hz, 1H), 7.46 (m, 1H), 7.43 (dd, *J*_1_=8.4 Hz, *J*_2_=7.8 Hz, 1H), 7.34 (m, 1H). ^13^C NMR (151 MHz, 1,1,2,2-tetrachloroethane-*d*_2_, 100 °C): δ 139.95, 134.19, 133.95, 130.04, 130.00, 128.10, 126.83, 124.74 and 122.69. (Two signals for the carbon atoms bonding to the boron atoms were not observed due to the quadrupolar relaxation of the boron atom); ^11^B-NMR (128 MHz, 1,1,2,2-tetrachloroethane-*d*_2_, 100 °C): δ 69.3; HRMS (*m*/*z*):[M]^+^ calcd for C_40_H_24_B_2_Br_2_: 686.0425; found, 686.0433.

### X-ray crystallographic analysis

Single crystals of **1** were obtained by slow diffusion of octane into a solution of **1** in chlorobenzene (Supplementary Fig. 11b). The intensity data were collected at 123 K with a Rigaku X-ray diffractometer equipped with a molybdenum FR-X microfocus generator, VariMax-Mo optics and a PILATUS 200 K detector. In total 22,714 reflections were collected, among which 5,303 reflections were independent (*R*_int_=0.0410). The structure was solved by direct methods (SHELXS-2013) and refined by full-matrix least-squares on *F*^2^ for all reflections (SHELXL-2013). All non-hydrogen atoms were refined anisotropically and all hydrogen atoms were placed using AFIX instructions. The crystal data are as follows: Formula C_40_H_24_B_2_Br_2_; FW=686.03, monoclinic, crystal size 0.14 × 0.02 × 0.02 mm^3^, *C*2/*c*, *a*=19.259(7) Å, *b*=17.907(6) Å, *c*=18.056(6) Å, *β*=102.721(4) Å, *V*=6,074(4) Å^3^, *Z*=8, *D*_calcd_=1.500 g cm^−3^; *R*_1_=0.0396 (*I*>2σ(*I*)), *wR*_2_=0.1006 (all data), GOF=0.987.

### Thermogravimetric analysis

Thermogravimetric analysis was performed on a Seiko EXSTAR 6,000 TG/DTA 6,200 apparatus at a heating rate of 5 °C min^−1^ under a nitrogen atmosphere (Supplementary Fig. 11c).

### Characterization of precursor molecule

NMR spectra of the precursor **1** are shown in Supplementary Fig. 11d. ^1^H and ^13^C NMR spectra were recorded with a JEOL A-600 spectrometer (600 MHz for ^1^H and 151 MHz for ^13^C). ^11^B-NMR spectrum was recorded with a JEOL AL-400 spectrometer (128 MHz for ^11^B). Chemical shifts are reported in δ ppm with reference to residual protons and carbons of 1,1,2,2-tetrachloroethane-*d*_2_ (δ 6.00 ppm in ^1^H-NMR and δ 73.78 ppm in ^13^C NMR). The external standard of BF_3_·OEt_2_ was used for ^11^B NMR. The mass spectrum was measured with a Bruker Daltonics micrOTOF Focus using a positive-mode Atmospheric Pressure Chemical Ionization (APCI)-time of flight method in a CHCl_3_ solution. Thin layer chromatography was performed on plates coated with 0.25-mm thickness of silica gel 60 *F*_254_ (Merck). Column chromatography was performed using silica gel PSQ 100B (Fuji Silysia Chemical).

### Theoretical calculation

All first principles calculations in this work were performed using the periodic plane-wave basis Vienna ab-initio simulation package (VASP) code^39^,^40^ implementing the spin-polarized DFT. To accurately include van der Waals interactions in this system we used the optB86P functional^41^,^42^,^43^. Projected augmented wave potentials were used to describe the core electrons^44^. A kinetic energy cutoff of 550 eV was found to converge the total energy of all the systems to within 10 meV. All STM simulations were done using the basic Tersoff–Hamann approach^45^. The properties of the bulk and surface of Au and graphite, and a NO molecule, were carefully checked within this methodology, and excellent agreement was achieved with experiments. Systematic *k*-point convergence was checked for all systems and a 5 × 1 × 1 mesh was found to be sufficient for the large GNR-Au systems. All reference calculations for adsorption energies, charge densities and projected density of states were performed with 4-anthracene GNR on gold, which had a lattice mismatch of only 1.4% (accommodated in the ribbon). When adding boron (B-GNR), this mismatch reduced to 0.3% as the ribbon expands slightly (Supplementary Fig. 4). To have a comparable anthracene separation of B–B sites as in experiments, particularly for the STM simulations, we also performed calculations on isolated 3-anthracene GNRs (for this size the mismatch with the Au surface would be over 10% unless an unfeasibly large unit cell is used). The simulated STM images were very similar, and hence were used for the image comparison in Fig. 1 for simplicity.

### Calculated AFM image

Recently, several mechanical AFM models have been proposed^46^,^47^, where the approach of a CO-functionalized AFM tip over a substrate can be simulated To obtain an accurate 3D force map. Key to these models is that during approach they allow the CO molecule to tilt under influence of the forces it experiences. This tilting proved to be key to understand the sharp contrast observed in atomic bonds. Here we used the model developed by Hapala *et al.*^46^ and extended it by adding electrostatic interactions on top of the van der Waals interactions. The B-GNR structure was taken from the DFT simulations of B-GNR on Au and the charges were extracted from the DFT calculations via Bader analysis^48^. The mechanical AFM model relies on empirical Lennard–Jones parameters, which were taken from the Chemistry at HARvard Molecular Mechanics (CHARMM) force field^49^, with additional boron parameters^50^. All other parameters are the same as intended by Hapala *et al.*, and the simulated AFM scan is performed at a resolution of 0.025 Å (in all directions), with a force tolerance criterion of 4 × 10^−6^ eV Å^−1^. The 3D force field is subsequently converted into a frequency shift image^51^, using the experimental parameters (*f*=24.76 kHz, *A*=53 pm and *k*=1,800 N m^−1^). In Fig. 3d the simulated AFM image for the approximate same height as in the experiments is shown (the height in the bottom left corner of the figure denotes the distance of the carbon of the CO-molecule to the plane going through the carbons of the B-GNR).

## 

## Additional information

**How to cite this article:** Kawai, S. *et al.* Atomically controlled substitutional boron-doping of graphene nanoribbons. *Nat. Commun.* 6:8098 doi: 10.1038/ncomms9098 (2015).

## Supplementary Material

Supplementary Information Supplementary Figures 1-11 and Supplementary References
