# Supplementary material for: Atomically controlled substitutional boron-doping of graphene nanoribbons
Source: Nat Commun. 2015 Aug 25;6:8098. doi: 10.1038/ncomms9098 (PMC4560828; doi:10.1038/ncomms9098)
Supplement: Supplementary Information — Supplementary Figures 1-11 and Supplementary References [file ncomms9098-s1.pdf]

Supplementary Information:

Supplementary Figures

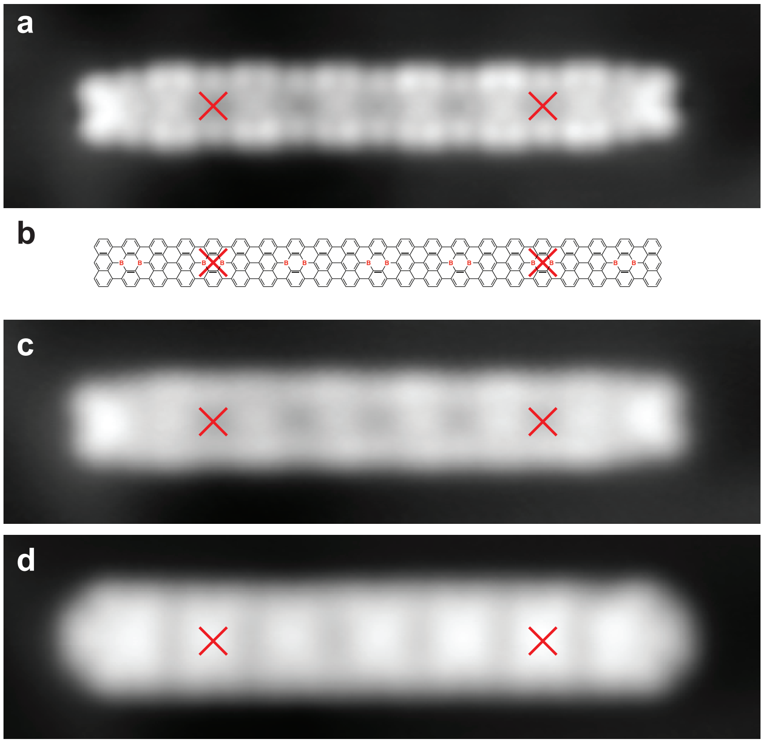

Supplementary Figure 1. **Isolated single B-GNR.** (**a**) STM topography of a single isolated B-GNR obtained with bias voltage of −5 mV (100 pA). As observed in Figure 1g, two different contrasts of the armchair type edge are observed. The darker and bright spots correspond to the hydrogen sites of the boron-doped and undoped anthracene moieties, respectively. We found that the contrast of the short zig-zag edge differs from that in Supplementary Fig. 7a and the protrusion at the center of the edge is missing. This contrast indicates the different structure of the B-GNR terminus. (**b**) Corresponding chemical structure judged by assigning the boron doped anthracene moieties. The contrast at the terminus of the short zigzag edge is distinct from that of pristine GNR, [1] indicating the significant influence of the boron doping on the terminus. This situation is different in the case of the defective terminus with an extra anthracene unit (see Supplementary Fig. 7). (**c**,**d**) By changing the bias voltage, the contrast at the boron site was changed, which is consistent with the observation in **c**, 3 V (50 pA), and **d**, −3 V (20 pA).

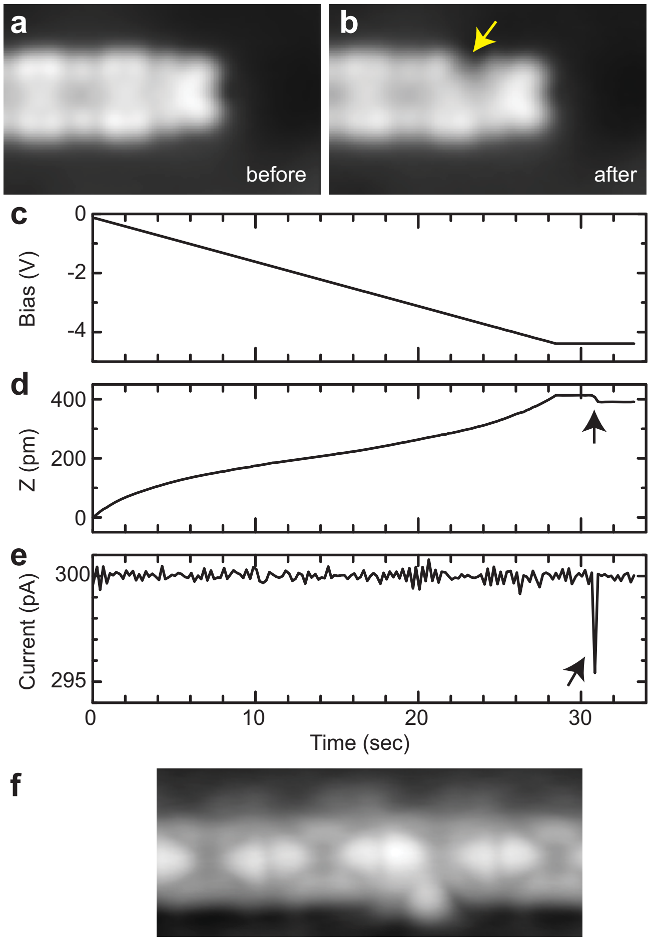

Supplementary Figure 2. **Tip-induced dehydrogenation.** (**a**,**b**) STM topographies before and after the tip-induced dehydrogenation at the armchair edge with a large negative tip bias voltage (typically −3.0 V, 20 pA). The arrow shows the missing hydrogen site. (**c**-**e**) Simultaneously recorded swept bias voltage, controlled tip-sample Z separation, and tunneling current (error signal) as a function of time, respectively. The tip was positioned at the maxima at the armchair edge (hydrogen site). The bias voltage was gradually increased to −4.390 V and kept until the dehydrogenation was caused. When this dehydrogenation happened, a relatively large error signal of the tunneling current was observed and a small shift of the tip-sample Z separation (22 pm) was detected. This method is repeatable. (**f**) STM topography, in which almost all hydrogens at the armchair edge were removed. The boron site became pronounced. The radical most probably connects to the Au atom of the substrate. Measurement parameter: $V = -5$ mV and $I = 100$ pA in **a**, $V = -2$ mV and $I = 20$ pA in **b**, and $V = -2$ mV and $I = 200$ pA in **f**.

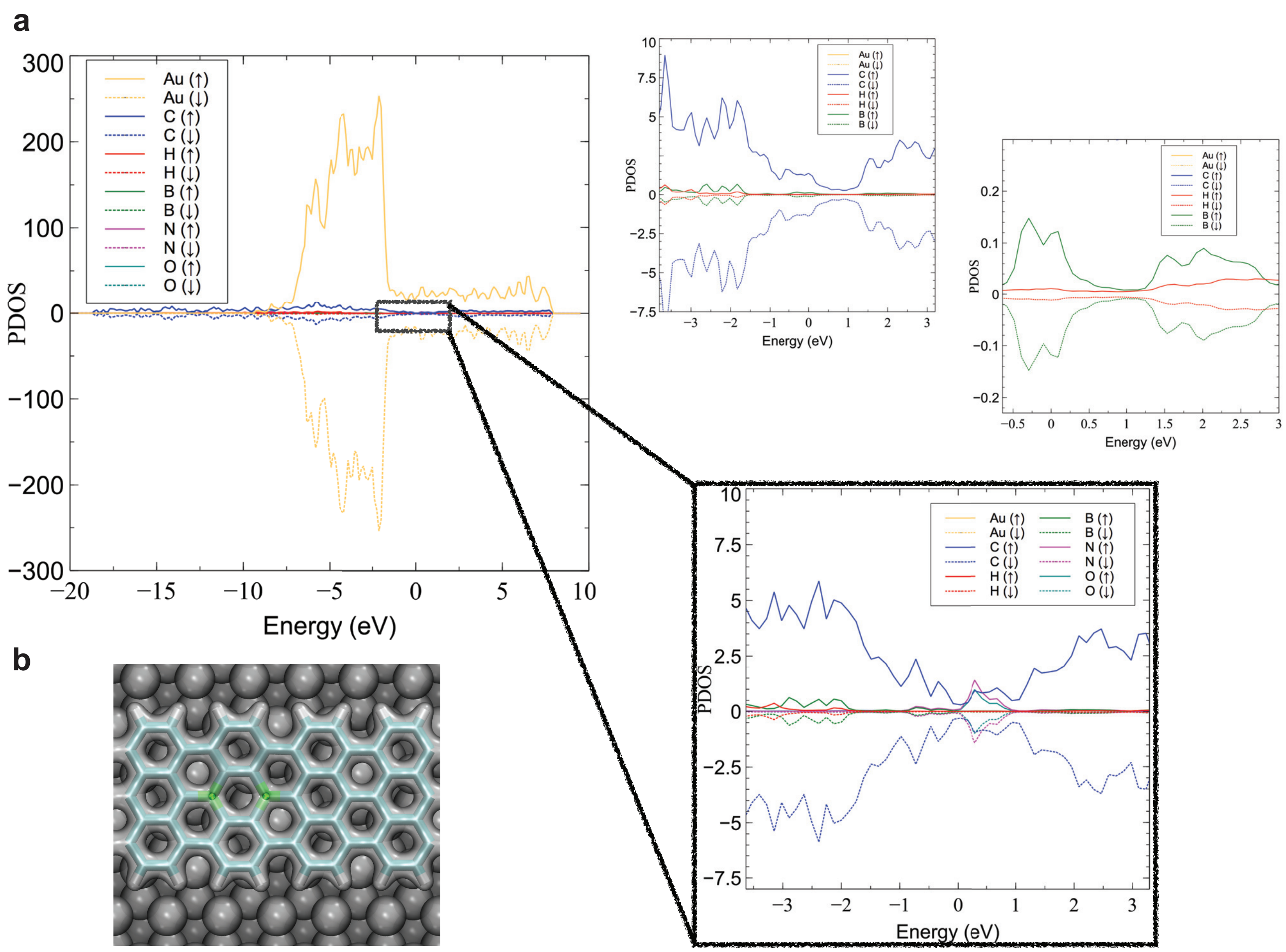

Supplementary Figure 3. **Calculated projected density of states.** (**a**) Calculated projected density of states (PDOS) for the B-GNR with and without an adsorbed NO molecule on the surface. The spin decomposition shows that there is very little spin polarisation in the system, in contrast to results for zigzag H-terminated nanoribbons [2]. The general PDOS features reproduce those seen in previous studies of armchair GNRs and B-GNRs [3], demonstrating semiconducting properties and a small band gap of around 1.5 eV (similar to previous DFT studies [4]). The NO states are hybridised with the C/B states near the Fermi energy, indicating the formation of covalent bonds and corresponding with the significant adsorption energy calculated. (**b**) Calculated valence charge density isosurface (in gray at 0.5 $e/\text{Å}^3$) of B-doped GNR on the Au substrate. The density is overlayed onto the bond structure of B-GNR on the Au substrate (Au bonds are not shown, C is light blue, B is green and H is white). The acceptor nature of the B-B defect can be seen by the "holes" in the valence charge density at the B sites, although, as discussed in Supplementary Fig. 7, the central position of the dopant means the electronic structure is similar to that of an ideal GNR.

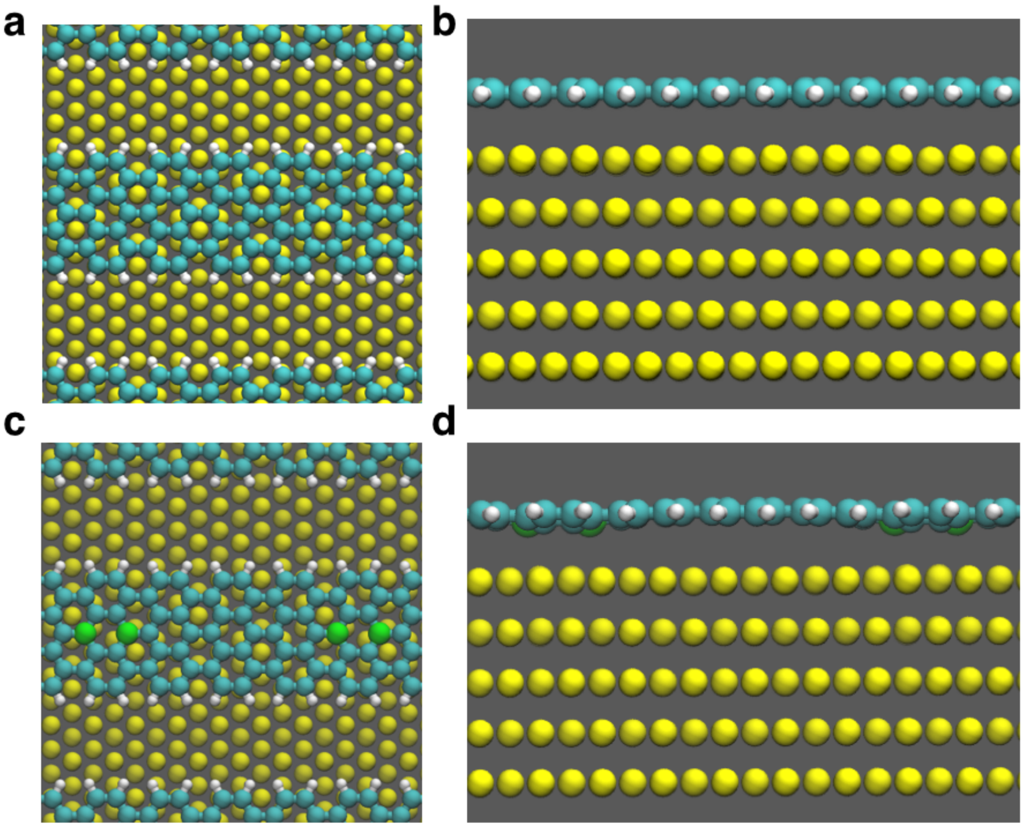

Supplementary Figure 4. **Flatness of B-GNR.** (**a**,**b**) Calculated geometry of an undoped GNR and (**c**,**d**), a B-doped GNR having three pristine anthracene units between the boron sites (see Methods). It was previously reported that the boron dopant in graphene strongly interacts with the Cu substrate because the localized empty P_z state attracts the boron site to the Cu substrate by 39 pm.[5] We see a similar deformation with respect to the undoped ribbon of around 30 pm on the Au substrate for both boron sites and the nearest neighbour carbons. Note also that undoped regions between the boron sites of the doped nanoribbon bend away from the pinned boron sites by around 40 pm and are 10 pm further from the Au substrate than the fully undoped ribbon in this model.

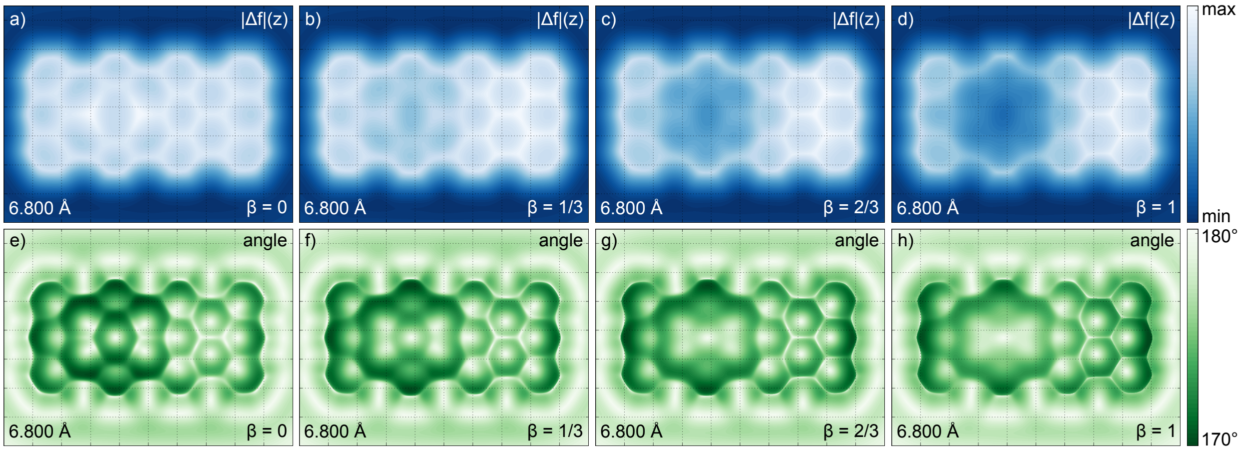

Supplementary Figure 5. **Effect of B-GNR distortion on simulated AFM images.** (**a**-**d**) Frequency shift maps and (**e-f**) the out of plane bending of the CO-molecule, calculated with different magnitudes of the artificially-changed topographic distortion, i.e., the dip of the B-B site with respect to the plane of the GNR. The magnitude of the dip of B-B, related nearby atomic displacements, was scaled (using parameter β) from the DFT results ($\beta = 1$, and a dip of 30 pm) to a completely flat B-GNR ($\beta = 0$, dip is 0 pm) with intermediate stages of $\beta = 1/3, 2/3$. It is clear that in the absence of the distortion the borons cannot be identified by a clear change in contrast as shown in **a**, whereas they easily can be seen in the presence of the distortion as shown in **d**. On the bottom row the tilting of the CO-tip is shown to be effected by the distortion as well (an angle of 180° means the CO is pointing down), making it easy to see the individual rings in the absence of the distortion, which is much harder around the borons when the distortion is present. **d** is the same as Figure 3d.

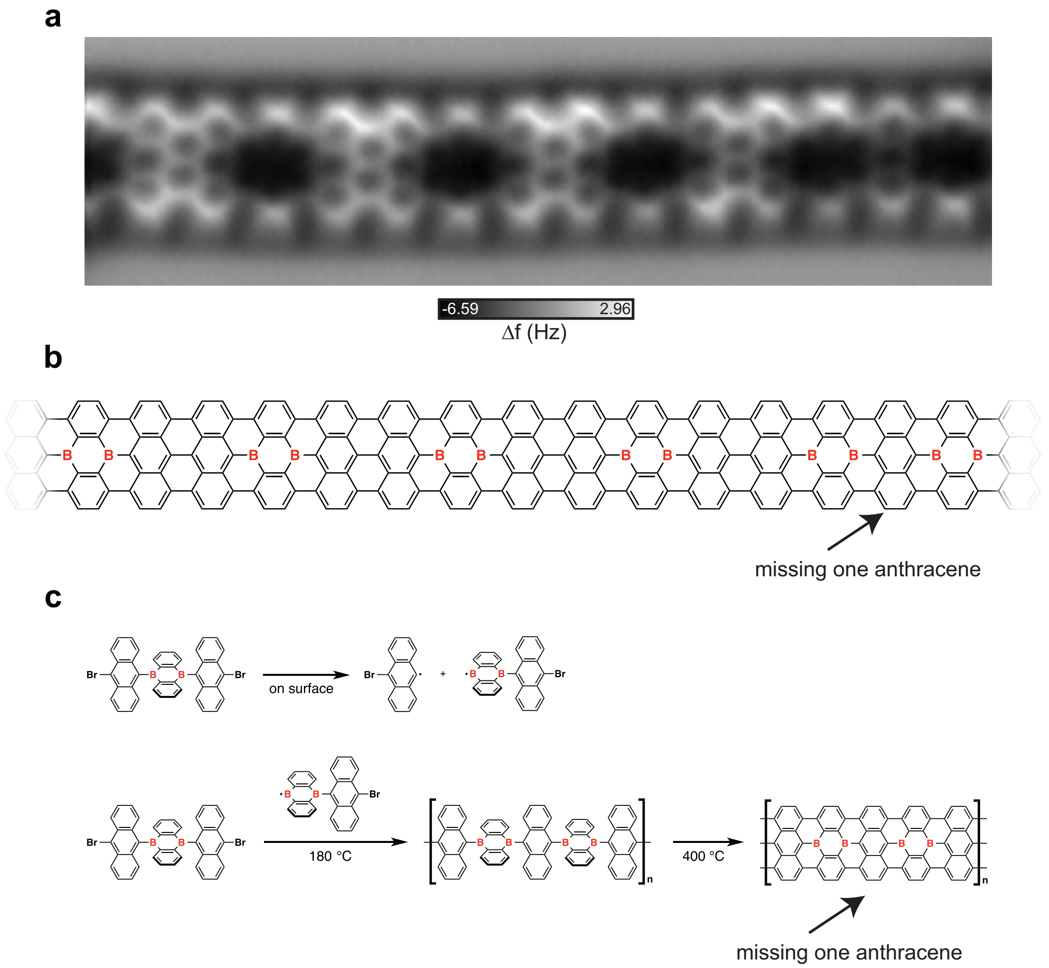

Supplementary Figure 6. **Defect of B-GNR.** (**a**) AFM image of N=7 B-GNR with a defect. At the right hand side, one pristine anthracene unit was missing. (**b**) Corresponding chemical structure. (**c**) Plausible chemical reaction path. The presence of the defective precursor molecules cannot be excluded, but most probably the B-C bond in the precursor molecule is cracked on the surface in the first reaction. Measurement parameters: $A = 60$ pm and $V_{\mathrm{bias}} = 0$ V.

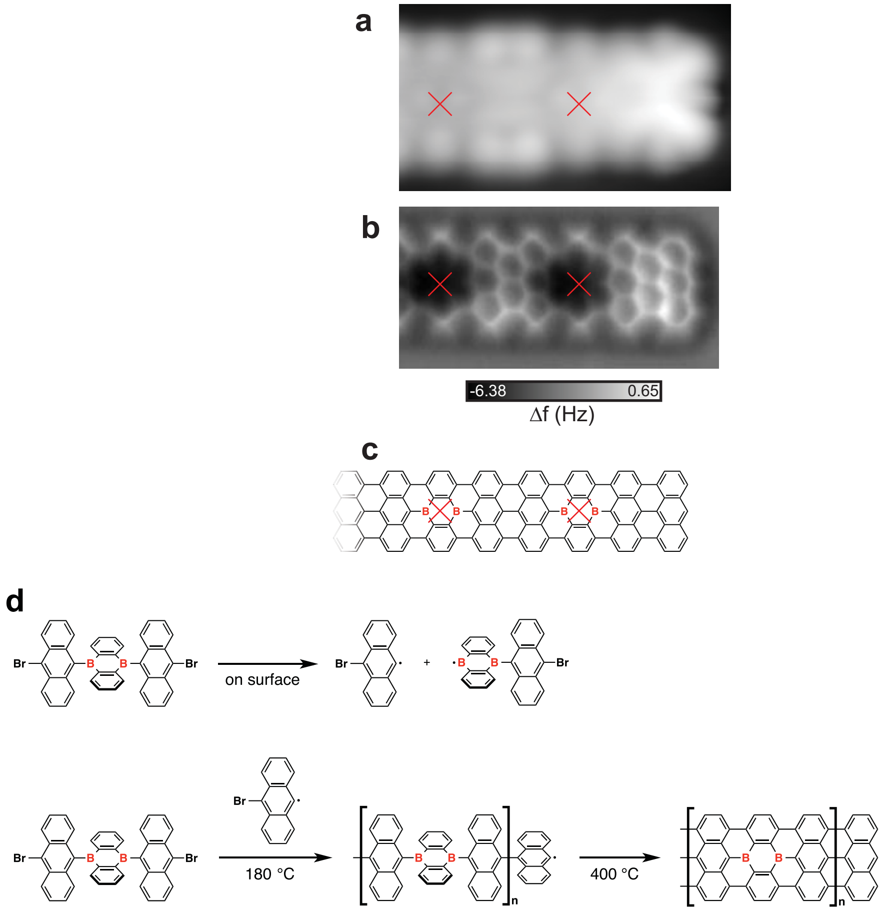

Supplementary Figure 7. Defect at the terminus of a B-GNR. (**a**) STM topography of the B-GNR terminus. Beside the contrast arising from the boron dopant, the STM topographic contrast at the short zig-zag edge is quite similar to that observed in the pristine GNR, namely the head of an insect. [1] Thus, the electronic state at the edge should be the same to that of pristine GNR. This observation is in agreement with the previous theoretical study, and our own calculations, in which the electronic structure at the zig-zag edge GNR is only sensitive to the boron atoms doped very close to the edge. [6] (**b**) Corresponding AFM image, obtained at the same type of edge with a CO tip of AFM. The short zig-zag edge was terminated with the two anthracene moieties. (**c**) Chemical structure. In the designed chemical reaction (Fig. 1a), the terminus is composed of one anthracene moiety. Thus, the B-GNR observed in **a,b** is defective. (**d**) Plausible chemical reaction path. In contrast to the reaction in Supplementary Fig. 6c, 9-bromoanthracene radical may take part in the reaction. Measurement parameters: $V = -2$ mV and $I = 500$ pA in **a** and $V = 0$ mV and $A = 70$ pm in **b**.

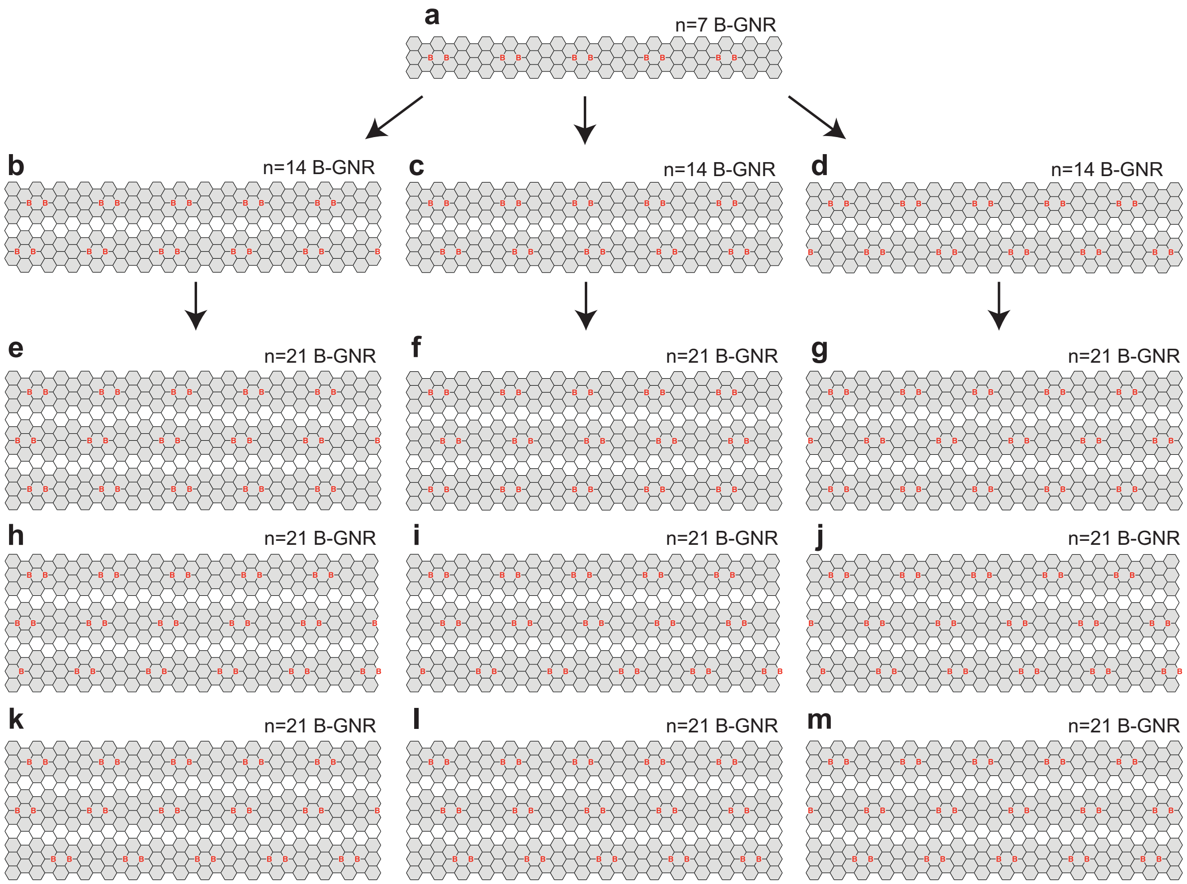

Supplementary Figure 8. **Chemical structures of N=7, 14, and 21 B-GNR** (**a**) Chemical structure of N=7 B-GNR. By annealing at above 400 °C, the graphene nanoribbon (GNR) on Au(111) can be fused and the width becomes larger stepwise [7]. In contrast to the pristine GNR [8], the B-GNR has two boron atoms in every three anthracene moieties and thus abundant structures can be found. (**b-d**) Fused N=14 B-GNR. Since the alignment of the boron-doped anthracene cannot be controlled in the fusion process, N=14 B-GNR has three different structures as shifted phase (**b, c**) and as out-of-phase (**d**). The structures of the shifted phase are mirror images of each other. The variety of the structure increases by the i number of the fused N=7 B-GNR as 3^{i-1} (**e-m**) Fused N=21 B-GNR. **g** shows the perfectly ordered out-of-phase N=21 B-GNR, which is experimentally observed in Figure 3g.

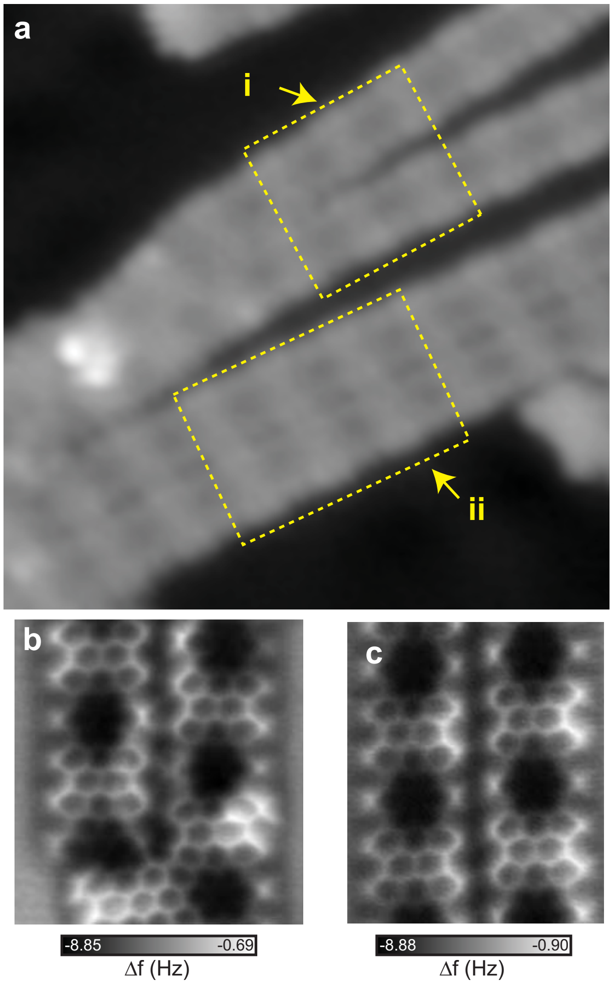

Supplementary Figure 9. **Fusing B-GNR.** (**a**) STM topography, showing partially fused GNRs. At the top, two B-GNRs are connected and at the bottom, two B-GNRs run parallel to each other. (**b**) AFM image, taken with a CO terminated tip at the area indicated by **i** in **a**. At the bottom, two GNRs are connected but at the upper part, they are not fused. Since the repulsion of the hydrogen atom at the armchair edge induces the stress at the contact, there is a defect at the left B-GNR. (**c**) AFM image, taken at the **ii** area indicated in **a**. Two B-GNRs runs in parallel. Due to the repulsion between two hydrogen atoms, the B-GNRs are shifted by half size of the benzene ring. Measurement parameters: $V = -200$ mV and $I = 1$ pA in **a**, $V = 0$ V and $A = 70$ pm in **b, c**.

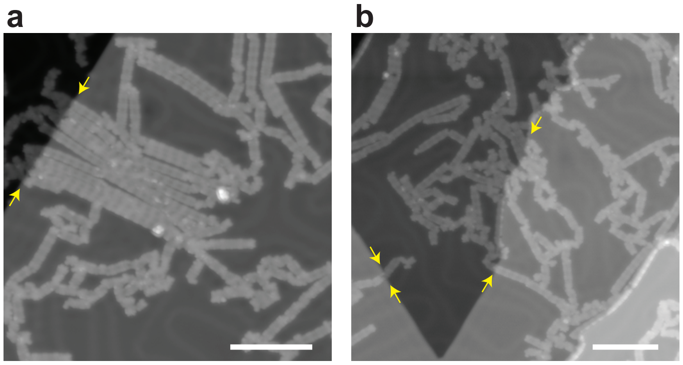

Supplementary Figure 10. **Diffusion of Au step edges under B-GNR.** (**a**,**b**) Examples of a defused Au step edge below the B-GNRs. In the fusion process, we annealed the Au(111) substrate at 510 °C, which is about 50 °C higher than the standard sample cleaning process (argon sputtering and annealing at 450 °C). Thus, the step of Au(111) diffuses at this temperature. B-GNRs form across the steps of Au(111). Since such B-GNRs are not observed in the standard annealing process at 400 °C (Fig. 1d), Au steps move underneath B-GNRs. Interestingly, no defect, relating to this process, was observed, which is another evidence that the doped boron atoms are highly stable. Scale bar, 10nm. Measurement parameters: $V = -200$ mV and $I = 1$ pA.

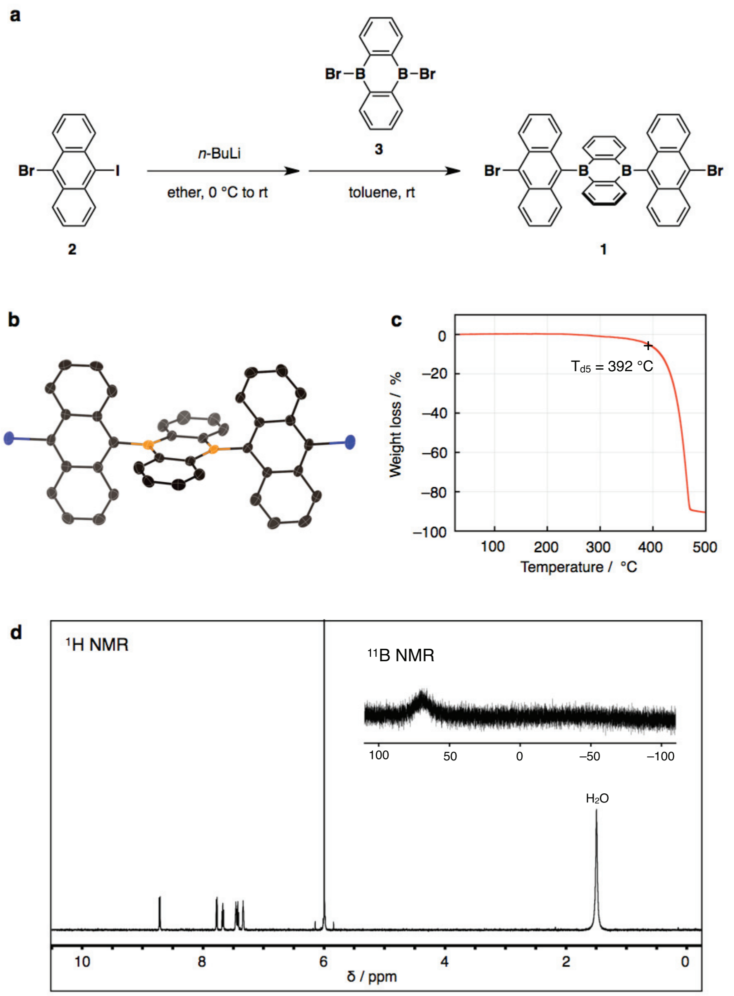

Supplementary Figure 11. **Synthesis and characterization of B-GNR precursor 1.** (**a**) Synthetic scheme of **1**. The precursor **1** was synthesized from the reaction of **2** with n-BuLi followed by the treatment with **3**. Details are described in the Methods section. (**b**) X-ray crystal structure of **1**. The thermal ellipsoids are drawn at the 50% probability level. Black: carbon, orange: boron, blue: bromine. The terminal anthracene units and the 9,10-dibora-9,10-dihydroanthracene moiety are orthogonal to each other due to the steric repulsion of the hydrogen atoms. (**c**) Thermogravimetric analysis of **1** by heating at 5 °C min^{-1} under a nitrogen atmosphere. The precursor **1** is thermally stable up to 250 °C in an aluminum pan. 5% weight loss was observed at 392 °C. (**d**) ^{1}H NMR spectrum of **1** in 1,1,2,2-tetrachloroethane-d_2 at 100 °C. Inset: ^{11}B NMR spectrum of **1** in 1,1,2,2-tetrachloroethane-d_2 at 100 °C. The chemical shifts of these spectra as well as ^{13}C NMR spectrum are presented in the Methods section. A typical broad signal for tri-coordinated boron was observed in the ^{11}B NMR spectrum.

SUPPLEMENTARY REFERENCES

[1] Talirz, L. *et al.* Termini of bottom-up fabricated graphene nanoribbons. *J. Am. Chem. Soc.* **135** 2060–2063 (2013).

[2] Li, Y., Zhang, W., Morgenstern, M. & Mazzarello, R. Electronic and magnetic properties of zigzag graphene nanoribbons on the (111) surface of Cu, Ag, and Au. *Phys. Rev. Lett.* **110**, 216804 (2013).

[3] Yu, S. S., Zheng, W. T. & Jiang, Q. Electronic properties of nitrogen-/boron-doped graphene nanoribbons with armchair edges. *IEEE Trans. Nanotechnol.* **9**, 78–81 (2010).

[4] Ruffieux, P. *et al.* Electronic Structure of Atomically Precise Graphene Nanoribbons. *ACS Nano*, **6**, 6930–6935 (2012).

[5] Zhao, L. *et al.* Local atomic and electronic structure of boron chemical doping in monolayer graphene. *Nano Lett.* **13**, 4659–4665 (2013).

[6] Biel, B., Blase, X., Triozon, F. & Roche, S. Anomalous doping effects on charge transport in graphene nanoribbons. *Phys. Rev. Lett.* **102**, 096803 (2009).

[7] Huang, H. *et al.* Spatially resolved electronic structures of atomically precise armchair graphene nanoribbons. *Sci. Rep.* **2**, 983 (2012).

[8] Cai, J. *et al.* Atomically precise bottom-up fabrication of graphene nanoribbons. *Nature* **466**, 470–473 (2010).
